# Bite force production and the origin of *Homo*

**DOI:** 10.1098/rsos.241879

**Published:** 2025-04-23

**Authors:** Justin A. Ledogar, Stefano Benazzi, Amanda L. Smith, Paul C. Dechow, Qian Wang, Rebecca W. Cook, Dimitri Neaux, Callum F. Ross, Ian R. Grosse, Barth W. Wright, Gerhard W. Weber, Craig Byron, Stephen Wroe, David S. Strait

**Affiliations:** ^1^ Department of Biomedical Health Sciences, East Tennessee State University, Johnson City, TN, USA; ^2^ Department of Cultural Heritage, University of Bologna, Bologna, Emilia-Romagna, Italy; ^3^ Department of Fundamental Biomedical Sciences, Touro University California, Vallejo, CA, USA; ^4^ Department of Biomedical Sciences, Texas A&M College of Dentistry, Dallas, TX, USA; ^5^ Department of Physiology and Anatomy, University of North Texas Health Science Center at Fort Worth, Fort Worth, TX, USA; ^6^ Department of Evolutionary Anthropology, Duke University, Durham, NC, USA; ^7^ Archéozoologie, Archéobotanique: Sociétés, Pratiques et Environnements, Muséum National d'Histoire Naturelle, Paris, France; ^8^ Department of Organismal Biology and Anatomy, University of Chicago, Chicago, IL, USA; ^9^ Department of Mechanical and Industrial Engineering, University of Massachusetts, Amherst, MA, USA; ^10^ Department of Surgery, University of Kansas Medical Center, KS, USA; ^11^ Department of Evolutionary Anthropology, University of Vienna, Vienna, Austria; ^12^ Human Evolution and Archaeological Sciences, University of Vienna, Vienna, Austria; ^13^ Department of Biology, Mercer University, Macon, GA, USA; ^14^ Department of Zoology, University of New England, Armidale, New South Wales, Australia; ^15^ Department of Anthropology, Washington University in St Louis, St Louis, MO, USA; ^16^ Palaeo-Research Institute, University of Johannesburg, Auckland Park, South Africa; ^17^ DFG Center for Advanced Studies ‘Words, Bones, Genes, Tools’, University of Tübingen, Tubingen, Baden-Württemberg, Germany

**Keywords:** human evolution, australopiths, diet, finite element analysis, *Homo habilis*

## Abstract

The divergence of *Homo* from gracile australopiths has been described as a trend of decreasing dentognathic size and robusticity, precipitated by stone tool use and/or a shift to softer foods, including meat. Yet, mechanical evidence supporting this narrative is sparse, and isotopic and archaeological data have led to the suggestion that a shift away from a gracile australopith-like diet would not have occurred in the most basal members of *Homo* but rather only with the appearance of *Homo erectus,* implying that the origin of our genus is not rooted in dietary change. Here, we provide mechanical evidence that *Homo habilis* exhibits an australopith-like pattern of facial strain during biting but, unlike most australopiths, was not suited for a diet that required forceful processing by the molar teeth. *Homo habilis* was at elevated risk of distractive jaw joint forces during those bites, constraining muscle recruitment so as to avoid generating uncomfortable/dangerous levels of tension in the joint. Modern humans have similar limitations. This suggests that selection on skeletal traits favouring forceful postcanine processing was relaxed by the earliest stages in the evolution of our genus, implying that dietary or food processing changes played an important role in the emergence of *Homo*.

## 1. Introduction

Gracile australopiths are variably characterized by a suite of derived dentognathic features that either structurally reinforce the facial skeleton (e.g. [1,2]), improve the efficiency of bite force generation (e.g. [3–5]), or otherwise enhance the ability of the feeding apparatus to process foods that were mechanically challenging to eat (e.g. [6–8]). The genus *Homo* is almost certainly descended from a gracile australopith ancestor (e.g. [9–13]), so the variably expressed loss of australopith features leads naturally to the hypothesis that the feeding apparatus of basal *Homo* was comparatively weak and less efficient, perhaps related to extra-oral food processing or a shift to a less demanding diet that may have included meat (see review in Ungar *et al.* [14]). Yet, current thought on hominin facial evolution [15] challenges the conventional wisdom that dentognathic reduction in earliest *Homo* corresponds to the advent of tool-assisted meat consumption and its concomitant lowering of feeding forces; rather, the key dietary shift in *Homo w*ould have occurred later, in *Homo erectus.* Evidence in support of this view, and against the traditional interpretation, is that the earliest stone tools [16,17] precede the earliest members of the genus *Homo* (e.g. [18,19]) by several hundred thousand years, that the diets of basal species *Homo habilis* and *Homo rudolfensis* isotopically resemble those of gracile australopiths (e.g. [20–22]), and that the jaws, teeth and faces of basal *Homo* are not, in fact, truly reduced [15]. A key obstacle in evaluating competing hypotheses regarding diet and the origin of *Homo* is that the mechanical premises of these inferences are difficult to test. Here, we use finite element analysis (FEA), a computer-assisted engineering technique (e.g. [23–26]), to simulate the mechanics of biting in *H. habilis* and test whether these mechanics were australopith-like or like later members of the genus *Homo*.

We analysed biting efficiency and structural strength during premolar (P^3^) and molar (M^2^) biting in a newly created finite element model (FEM) of *H. habilis* based on KNM-ER 1813 (a fossil specimen without derived australopith facial features), and compared the mechanical results with those from previously constructed [5,27–30] models of ‘gracile’ and ‘robust’ australopith species (*Australopithecus afarensis, A. africanus, A. sediba, Paranthropus boisei*), modern chimpanzees (*Pan troglodytes*), and recent modern humans (*H. sapiens*). For each bite case, we subjected each FEM to isometrically scaled muscle forces derived from a chimpanzee (*P. troglodytes*) simulating a maximal bite (all muscles contracting at peak activity). This removes size as a confounding variable and ensures that stress and strain differences between the models are exclusively a function of differences in shape [31]. This modelling experiment provides a powerful means of assessing the relative strength of each model, as structural strength is inversely proportional to stress, which is in turn related to strain. A complication, however, is that jaw adductor muscles are differently proportioned and relatively smaller in recent modern humans than in chimpanzees (see also Eng *et al*. [4]), meaning that the human FEMs are being subjected to abnormally high loads. Moreover, we do not know whether it is more appropriate to load the *H. habilis* FEM with scaled chimpanzee or modern human muscle forces. Thus, our assessment of structural strength may not provide the best assessment of bite force generation for all species in our sample. Accordingly, bite force generation was examined separately (see also Eng *et al.* [4]) by loading the chimpanzee and australopith crania with isometrically scaled muscle forces derived from a chimpanzee [32], and by loading the modern human crania with isometrically scaled muscle forces derived from a human [29]. The *H. habilis* cranium was loaded twice, once with muscle forces scaled from the chimpanzee baseline and once from the human baseline.

It is important to note that our assessment of feeding biomechanics focuses on static maximum bite force production as opposed to chewing and therefore does not capture the full range of mechanical performance in the craniofacial skeleton during a complete gape cycle. Recently, Panagiotopoulou *et al*. [33] found that dynamic FEMs that incorporate muscle force data from *in vivo* feeding experiments experience variation in strain magnitudes depending on the time point in the gape cycle being modelled. Such analyses can be more informative than static FEMs with respect to understanding how asymmetry in muscle activation patterns impacts loading regimes during mastication. However, while electromyographic (EMG) analyses of muscle activation patterns during chewing account for variation in the timing of peak activity between the working- and balancing-side jaw adductors, it is nonetheless a fact that muscle forces, bite forces, and stresses and strains during maximal biting represent an upper bound on the magnitude of those variables that can be produced at any given point during a feeding cycle. Thus, even though our model simulation does not capture the range of variation displayed by those variables during feeding, it nonetheless provides a limit that can be used to assess relationships between maximal bite force production and structural strength.

Moreover, given the fact that EMG activity in *H. habilis* is unknowable, and that patterns of muscle activity can be highly variable among individuals and even between chews of the same foods by a single individual [34–36], it is reasonable to model the upper limits of mechanical performance because all organisms possess such limits and thus the limits provide an apples-to-apples comparison between individuals in different taxa. Indeed, numerous studies have applied static models to examine maximum bite force and test evolutionary hypotheses in a wide range of living primate species (e.g. [37–42]), fossil hominins (e.g. [3,4,43]), and non-primate mammals (e.g. [44–46]). Although the production of bite forces that approach maximum potential may be a relatively uncommon behaviour, the selective importance of a behaviour may not necessarily be correlated with the frequency of that behaviour (e.g. [47,48]). For example, the exploitation of fallback foods that are consumed only when preferred foods are unavailable may involve processing food tissues that are mechanically challenging to eat [49–54], and yet the ability to eat those foods may be critical to survival.

Furthermore, Constantino *et al*. [55] found that some living primates and fossil hominins exhibit antemortem chips on their postcanine teeth that suggest the use of high bite forces. Notably, they found that orangutans are capable of generating 2500 N of molar bite force, which is comparable to food fracture experiments that demonstrate orangutans generate over 2000 N of force when cracking open macadamia nuts [56,57]. That amount of force is also sufficient to fracture *Mezzettia* seeds along their germination band, and consuming such seeds comprises 13.3%–18.2% of the average monthly feeding time of orangutans at the Sanbangau field area [58,59]. Robust capuchins, saki monkeys and sooty mangabeys are other examples of primates who rely periodically or frequently on the production of high bite forces in order to process hard foods [52,60–65]. Direct behavioural observations of such primates in the wild (e.g. [64–67]) suggest that the initial fracturing of hard foods may involve static bites, rather than dynamic masticatory movements, so there is reason to suspect that static biting may be a selectively important behaviour in these species. Finally, hard object feeding is a behaviour that has long been thought to be selectively important in australopiths (e.g. [3,27,68–70]) so it is reasonable to investigate whether *H. habilis* differs from australopiths with respect to this loading regime.

## 2. Results and discussion

**Figure 1.**
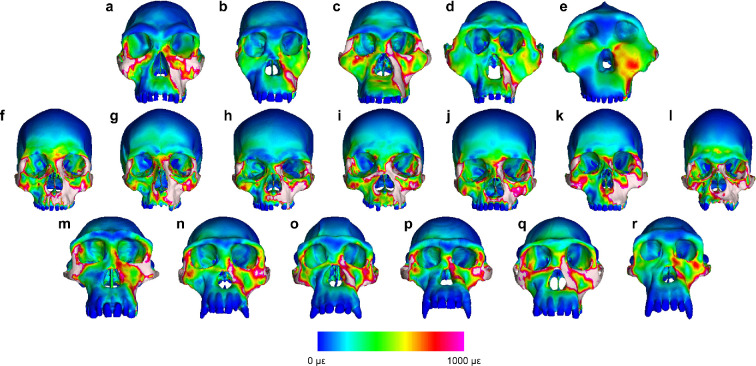
Colour mapping of von Mises strain in FEMs of (a) *Homo habilis*, (b) *Australopithecus sediba*, (c) *A. africanus*, (d) *A. afarensis*, (e) *Paranthropus boisei*, (f–l) recent modern humans and (m–r) modern chimpanzees during maximal bites on the upper third premolar (P^3^). Colours correspond to strain magnitude, with white indicating strains greater than 1000 microstrain (με). Chimpanzee crania were intentionally selected to be morphologically different from each other [28] and are labelled according to whether they represent the extreme positive or negative ends of the range of variation along three principal components of shape. Human crania were also intentionally selected to be morphologically different from each other [29] using their individual pairwise distances. One model (GRGL) was selected as an ‘average’ representative of human cranial shape based on its close proximity (i.e. small Euclidean distance) to the group centroid.

**Figure 2.**
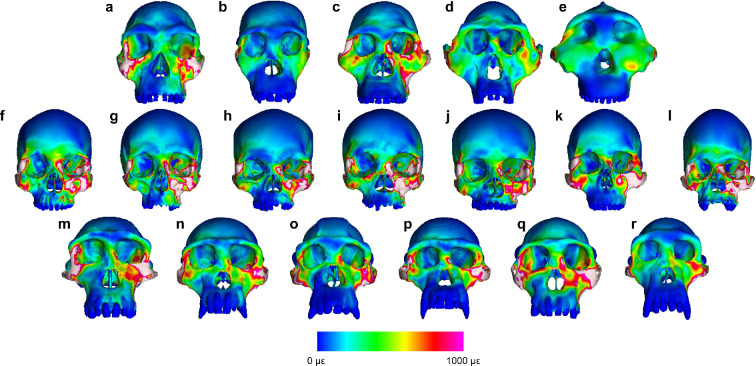
Colour mapping of von Mises strain in FEMs of (a) *Homo habilis*, (b) *Australopithecus sediba*, (c) *A. africanus*, (d) *A. afarensis*, (e) *Paranthropus boisei*, (f–l) recent modern humans and (m–r) modern chimpanzees during maximal bites on the upper second molar (M^2^). Colours correspond to strain magnitude, with white indicating strains greater than 1000 microstrain (με).

**Figure 3.**
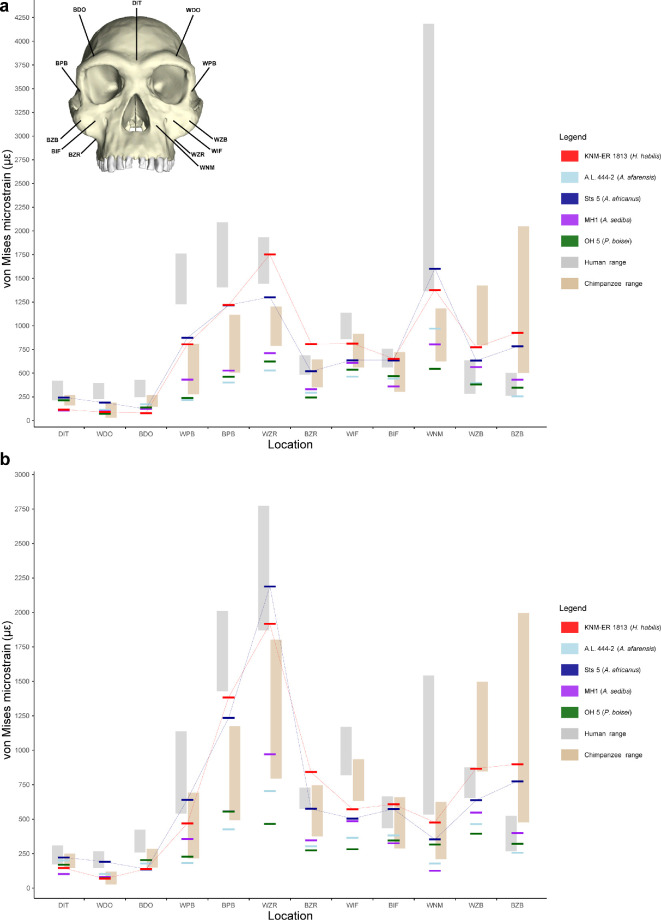
The von Mises strain magnitudes in microstrain (με) sampled from 12 facial sites during simulations of (a) P^3^ and (b) M^2^ biting in FEMs of fossil hominins, modern humans, and chimpanzees. The thin red and blue lines highlight the values for KNM-ER 1813 and Sts 5, respectively. DIT, dorsal interorbital; WDO, working dorsal orbital; BDO, balancing dorsal orbital; WPB, working postorbital bar; BPB, balancing postorbital bar; WZR, working zygomatic root; BZR, balancing zygomatic root; WIF, working infraorbital; BIF, balancing infraorbital; WNM, working nasal margin; WZB, working zygomatic body; BZB, balancing zygomatic body.

During both P^3^ and M^2^ biting (figures 1, 2 and 3), strain magnitudes tend to be higher in modern humans than in chimpanzees in most (but not all) facial regions, indicating that the facial skeleton is broadly weaker in the former than in the latter. Strains in australopiths generally overlap with the chimpanzee range of variation, with *A. africanus* specimen Sts 5 often exhibiting the highest values. Facial strains in *H. habilis* specimen KNM-ER 1813 are either higher than or nearly as high as those of *A. africanus* except in the supraorbital region (which may not be informative with respect to dietary adaptation [71]). Notably, KNM-ER 1813 exhibits the highest strain magnitudes at both zygomatic roots, infraorbital regions and zygomatic bodies among fossil taxa. Technically, one could infer that the mid-face of *H. habilis* is weaker than those of australopiths, but a close comparison between the strain magnitudes in KNM-ER 1813 and Sts 5 reveals a striking similarity between the two (figure 3), despite profound differences in facial architecture. Thus, a better characterization of the strains in *H. habilis* is that it is similar to, but lies at the edge of, the australopith strain pattern.

A more pronounced difference between the *H. habilis* and most australopith FEMs concerned bite force production, particularly during molar biting. All primates (and most mammals) are subject to a critical constraint on bite force production in that simple biomechanical principles [44] prevent them from using their jaw adductor muscles equally on both sides of the jaw during bites on their more distal teeth (see electronic supplementary material); were they to do so, they would risk damaging the soft tissues of their temporomandibular joint. The basic predictions of this constrained lever model of bite force production [44] have been corroborated by comparative [72], modelling [29,73], ontogenetic [74] and experimental analyses [75–79]. Notably, experiments in humans [80] demonstrate that forceful biting on the distal-most teeth is associated with pain in the working-side temporomandibular joint (TMJ) and concomitantly lower muscle activity levels. Importantly, this constraint sets limits on maximum bite force production during bites on distal teeth because the balancing (non-biting) side muscles often cannot be employed to their full extent. The fact that mammals do not routinely damage their temporomandibular joints during bites on their distal teeth is a testament to how effectively mechanisms (probably involving proprioception) have evolved to modulate the activity of the jaw adductors.

The *H. habilis* FEM was very efficient at producing bite force, especially when loaded with scaled forces using the chimpanzee baseline (table 1). Australopiths are also efficient in this regard but for different reasons. Australopiths increase the mechanical advantage (MA) of their jaw adductor muscles through the anterior placement of key muscle attachments (e.g. superficial masseter [1]). In contrast, *H. habilis* is efficient because its face is retracted, possibly related to strong cranial base flexion (see electronic supplementary material), such that the load arm of the bite force is proportionally short. The australopith configuration presents a mechanical challenge, because with more anteriorly positioned muscle attachments it becomes increasingly likely that during molar bites the vector resultant of all of the jaw adductor muscles can pass outside of (i.e. anteromedial to) the ‘triangle of support’ defined by the bite point and the TMJs [44,72,76] (see electronic supplementary material). If this were to occur, the mandibular condyle on the working (i.e. biting) side would be distracted from (i.e. dislocated out of) the TMJ, potentially causing pain and injury [44,72,76]. In most australopiths, this possibility is structurally mitigated by having tall faces that position the bite point well below the TMJs, thereby increasing the likelihood that the resultant passes through (i.e. within the boundaries of) the steeply inclined triangle [5,27,30]. *Homo habilis,* like other members of the genus *Homo* with retracted faces [29,81], is also at risk of joint distraction but, lacking as tall a face, must behaviourally mitigate this risk by more substantially reducing balancing side muscle force. This shifts the resultant towards the working side and within the triangle, but at the cost of reducing maximum bite force. All primates may reduce balancing side muscle force to some extent during molar biting [72] in order to reduce the risk of joint distraction, but the need to do so in *H. habilis* is especially acute (table 2). The only other FEM that required a comparable reduction in balancing side muscle force during molar biting is *A. sediba* specimen MH1, a potentially close phylogenetic relative of *Homo* [82,83] (note that although MH1 is a juvenile, it has been previously shown [5] that extrapolation of its growth into adulthood would not meaningfully change the relative placements of its teeth, malar root and temporomandibular joint, meaning that its developmental age is not biasing biomechanical results). Estimating maximum bite force in *H. habilis* is complicated by the wildly different muscle forces derived from chimpanzee vs. human baselines (see electronic supplementary material). However, the mere fact that the FEM of KNM-ER 1813 is so constrained with respect to the utilization of its available muscle force is evidence enough to conclude that *H. habilis* was not optimized to bite forcefully on its molar teeth, unlike most australopiths but similar to modern humans [29], as well as *Homo floresiensis* [81].

**Table 1.** Muscle forces applied to FEMs based on differences in model volume to the 2/3 power (see also electronic supplementary material, table S2) and resulting outputs for bite force (BF), mechanical advantage (MA), and working-side temporomandibular joint reaction force (WJRF). All forces are in newtons (N).

model	muscle group	muscle force	premolar bite			molar bite		
			BF	MA	WJRF	BF	MA	WJRF
*Australopithecus afarensis*	chimpanzee—symmetrical	3911	1641	0.42	616.0	2431	0.62	181.6
*Australopithecus africanus*	chimpanzee—symmetrical	2893	1178	0.41	454.8	1786	0.62	48.4
*Australopithecus sediba*	chimpanzee—symmetrical	2658	1043	0.39	310.7	1827	0.69	−154.8
	chimpanzee—asymmetrical^c^	2272	—	—	—	1557	0.69	1.7
*Paranthropus boisei*	chimpanzee—symmetrical	5176	2053	0.40	885.4	3895	0.75	57.9
*Homo habilis*	chimpanzee—symmetrical	2945	1273	0.43	384.0	2428	0.82	−233.6
	chimpanzee—asymmetrical^d^	2282	—	—	—	1880	0.82	12.4
	human—symmetrical	831	313	0.38	143.7	596	0.72	−18.6
	human—asymmetrical^e^	747	—	—	—	537	0.72	4.8
*Pan troglodytes* (PC1+)^a^	chimpanzee—symmetrical	2540	818	0.32	466.9	1251	0.49	136.6
*Pan troglodytes* (PC1-)	chimpanzee—symmetrical	2980	1107	0.37	429.2	1522	0.51	104.9
*Pan troglodytes* (PC2+)	chimpanzee—symmetrical	2408	924	0.38	345.8	1350	0.56	77.1
*Pan troglodytes* (PC2-)	chimpanzee—symmetrical	2536	999	0.39	308.8	1481	0.58	35.6
*Pan troglodytes* (PC3+)	chimpanzee—symmetrical	3146	1310	0.42	398.7	1908	0.61	26
*Pan troglodytes* (PC3-)	chimpanzee—symmetrical	3269	1244	0.38	373.9	1779	0.54	−12.7
	chimpanzee—asymmetrical^f^	3187	—	—	—	1736	0.54	17.81
*Homo sapiens* (GRGL)	chimpanzee—symmetrical	3965	1724	0.43	499.8	2570	0.65	−208.2
	chimpanzee—asymmetrical^g^	3469	—	—	—	2252	0.65	13.0
*Homo sapiens* (BERG)	chimpanzee—symmetrical	3637	1720	0.47	405.1	2599	0.71	−185.7
	chimpanzee—asymmetrical^c^	3092	—	—	—	2213	0.71	29.1
*Homo sapiens* (KSAN1)	chimpanzee—symmetrical	3353	1462	0.44	343.3	2080	0.62	−188.0
	chimpanzee—asymmetrical^c^	2850	—	—	—	1771	0.62	37.2
*Homo sapiens* (KSAN2)	chimpanzee—symmetrical	2804	1272	0.45	311.7	1895	0.68	−163.8
	chimpanzee—asymmetrical^c^	2384	—	—	—	1610	0.68	19.0
*Homo sapiens* (MALP)	chimpanzee—symmetrical	2986	1358	0.45	384.4	2118	0.71	−203.3
	chimpanzee—asymmetrical^g^	2538	—	—	—	1797	0.71	38.3
*Homo sapiens* (TIGA)	chimpanzee—symmetrical	4418	1941	0.44	564.1	2896	0.66	−107.6
	chimpanzee—asymmetrical^h^	4086	—	—	—	2678	0.66	33.9
*Homo sapiens* (WAFR)	chimpanzee—symmetrical	3567	1383	0.39	489.3	2146	0.60	−61.1
	chimpanzee—asymmetrical^i^	3389	—	—	—	2036	0.60	13.1
*Homo sapiens* (GRGL)^b^	human—symmetrical	1118	441	0.39	167.4	658	0.59	−11.7
	human—asymmetrical^i^	1062	—	—	—	625	0.59	9.0
*Homo sapiens* (BERG)	human—symmetrical	1026	439	0.43	147.7	663	0.65	−7.0
	human—asymmetrical^f^	1000	—	—	—	647	0.65	1.3
*Homo sapiens* (KSAN1)	human—symmetrical	946	378	0.4	121.8	538	0.57	−17.5
	human—asymmetrical^i^	898	—	—	—	511	0.57	0.1
*Homo sapiens* (KSAN2)	human—symmetrical	791	333	0.42	106.8	496	0.63	−18.9
	human—asymmetrical^h^	732	—	—	—	459	0.63	3.0
*Homo sapiens* (MALP)	human—symmetrical	842	344	0.41	131.1	537	0.64	−19.9
	human—asymmetrical^i^	800	—	—	—	510	0.64	1.0
*Homo sapiens* (TIGA)	human—symmetrical	1246	507	0.41	188.0	756	0.61	13.7
*Homo sapiens* (WAFR)	human—symmetrical	1006	341	0.34	149.4	529	0.53	12.6

^a^ Specimen used as baseline for scaling purposes (chimpanzee group).

^b^ Specimen used as baseline for scaling purposes (human group).

^c^ Balancing-side muscles reduced relative to the working side by 30%.

^d^ Balancing-side muscles reduced relative to the working side by 45%.

^e^ Balancing-side muscles reduced relative to the working side by 20%.

^f^ Balancing-side muscles reduced relative to the working side by 5%.

^g^ Balancing-side muscles reduced relative to the working side by 25%.

^h^ Balancing-side muscles reduced relative to the working side by 15%.

^i^ Balancing-side muscles reduced relative to the working side by 10%.

**Table 2.** The proportion of balancing side muscle force reduction required to remove tension (distraction) at the working side temporomandibular joint (TMJ) in FEMs of chimpanzees (*Pan troglodytes*), australopiths, *Homo habilis* and recent *H. sapiens*.

species	% reduction in balancing side muscle force using scaled chimpanzee muscle forces	% reduction in balancing side muscle force using scaled human muscle forces
*Pan troglodytes*	0%–5%	—
*Australopithecus afarensis*	0%	—
*Australopithecus africanus*	0%	—
*Australopithecus sediba*	30%	—
*Paranthropus boisei*	0%	—
*Homo habilis*	45%	20%
*Homo sapiens*	10%–30%	0%–15%

Stedman *et al.* [84] suggest that reductions in the size of the jaw adductor muscles over human evolution occurred before the evolution of modern body proportions. They identify a gene (*MYH16*) encoding the predominant myosin-heavy chain expressed in the jaw adductor muscles that became inactivated, resulting in a reduction in the size (and thus strength) of the jaw adductor musculature and resulting in pleiotropic effects on craniofacial morphology, between 2.7 and 2.1 Ma, near the age of the earliest *Homo* [19] (other workers place the inactivation closer to 5.3 Ma [85]). Therefore, it is possible that small muscle size in *H. habilis* is a reflection of this inactivation, and that basal *Homo* species and possibly their immediate ancestors were experiencing relaxed selection for traits related to high bite force production. Our mechanical results for *H. habilis* and *A. sediba* demonstrating a decreased ability to produce forceful molar bites are consistent with this hypothesis.

The increased risk of jaw joint distraction in *H. habilis* due to facial retraction and decreased reliance on forceful biting using the distal molars could explain a trend of molar size reduction throughout the evolution of *Homo* that disproportionally affected the third molar (the risk of distraction is greatest during bites on this tooth). Evans *et al.* [86] found that australopiths follow the default mammalian pattern for tooth size, governed by an activator–inhibitor mechanism, where the largest postcanine tooth is either the M3, or the M2 and M3 are equal in size. They found that *Homo* species mostly exhibit a pattern where the largest tooth shifts mesially and that the distal molars exhibit a tightly linked decrease in size that is most notable in the M3. Third molar reduction is variable in *H. habilis* [87–89], but present in KNM-ER 1813 [87], which served as the basis for our FEM. Notably, such reduction is present in the recently described 2.8 Ma fossil from Ledi-Geraru (LD 350-1) [19] which may represent the earliest known member of our genus. Variability in this trait in basal *Homo* may be influenced by varying degrees of facial retraction and concomitant differences in jaw joint mechanics.

Several studies have linked increased jaw adductor leverage with third molar reduction and agenesis in primates, including humans [43,90,91]. For example, seed-predating New World primates with adaptations for increased bite force leverage have relatively small third molar crowns and roots [90–92]. With an anterior shift of the muscles, biting on the third molar increases the chances of TMJ distraction by shifting the muscle resultant vector outside of the triangle of support (see electronic supplementary material) formed by the mandibular condyles and the bite point [44]. Thus, the functional area of the postcanine tooth row is reduced and the expected result is a reduction of molar occlusal surface area, particularly the third molar, or even agenesis of this tooth. Third molar agenesis is common in modern humans [93], which has been linked to the combination of high bite force leverage and TMJ distraction found to characterize human molar biting [29]. In particular, Spencer and Demes [43] suggest that specialized anterior tooth biting and increased jaw adductor muscle leverage may be related to the high incidence of third molar reduction and agenesis among modern Inuit [38] due to the increased risk of distraction when biting on this tooth.

Our results are consistent with evidence for increased postcanine occlusal relief and therefore shearing potential in basal *Homo* [7], and the inferred increased reliance on displacement-limited foods [94] that are compliant yet crack-resistant (including but not limited to fibrous foliage, ‘leathery’ fruit carps and/or meat; these foods are informally considered ‘tough’ but this term is mechanically imprecise when used in this context [94]). Such foods do not fracture under the application of high forces, but rather due to displacements of food tissues leading to the accumulation of high tensile stresses and associated energy [94]. Meat is an example of such a food, but our results do not allow us to conclude definitively that meat eating drove the evolution of feeding biomechanics in basal *Homo.* Nonetheless, our results potentially indicate that elevated MA due to facial retraction in early *Homo* may have been advantageous when engaging in cyclical loading of ‘tough’ foods because enhanced muscular efficiency may reduce the risk of muscle fatigue, but the processing of such foods does not depend on the generation of high bite forces. In contrast, stress-limited foods [94] that are both stiff and crack-resistant fail under the application of high loads (such foods are informally considered ‘hard’ [94] although, again, this term is not always used in a mechanically precise way). Many australopith dentognathic features are well suited to generate and resist high loads (e.g. [1–5,8,27,30,32,95]), so the inability in *H. habilis* and *A. sediba* to optimally recruit their jaw adductor muscles during molar bites suggests that, unlike most australopiths, they would have been limited in their ability to consume stress-limited foods unless they relied on tools to process those foods extra-orally.

Thus, whereas all of the fossil hominin taxa examined here could generate bite force efficiently, only some could generate absolutely high bite forces. Differing constraints on bite force magnitudes are compatible with a hypothesis of dietary and/or feeding differences between these taxa, and are incompatible with the hypothesis [15] that a reduced ability to orally process mechanically challenging foods evolved first in *H. erectus* rather than in more basal members of the *Homo* clade. Our results suggest that biomechanical constraints on molar bite force production and their concomitant dietary limitations were already in place by the emergence of *H. habilis*. This hypothesis is consistent with the broad overlap in postcanine and jaw size between *H. habilis* and *H. erectus* [96], suggesting a foraging or food processing shift had already occurred in the former. This shift, which occurred at or near the base of the *Homo* clade, evidently did not involve a change in the isotopic composition of the diet compared with that of australopiths [20–22]. However, isotopic signals do not necessarily reflect differences in food mechanical properties. For example, van Casteren *et al.* [97] found that forest versus savannah-dwelling chimpanzees ate foods that differed significantly in crack resistance and stiffness, despite both populations having diets dominated by C_3_ foods.

Note that the above interpretation is unaffected by the phylogenetic relationships of *A. sediba* (see Mongle *et al*. [83]). If *A. sediba* is not a close relative of *Homo* (e.g. [98])*,* then the constraint on molar bite force production probably evolved in parallel in *A. sediba* and the last common ancestor (LCA) of the *Homo* clade. If, on the other hand, *A. sediba* is the sister taxon of *Homo* (e.g. [82]), then the constraint would have evolved in the LCA of *A. sediba* and *Homo* (indeed, such a phylogeny implies that the exclusion of *A. sediba* from *Homo* is essentially arbitrary). In either scenario, feeding mechanics in the earliest members of our genus would have been notably different from that in most australopiths.

Finally, the observation that the face of *H. habilis* is only marginally less strong than those of australopiths despite the complete lack of bony reinforcements that so distinctively characterize the latter is compatible with the recently proposed mechanical compensation hypothesis [30]. This hypothesis states that facial reinforcement features in australopiths evolved to mechanically compensate for facial weakening caused by the evolution of traits that enhance the efficiency of bite force production. In this view, massively enlarged postcanine teeth in australopiths leads necessarily to long tooth rows, and thus the placement of some teeth far anterior to the temporomandibular joint. This lengthens the load arm of the jaw apparatus, but bite forces on the more mesial cheek teeth (i.e. the premolars) can be maintained by modifying the facial skeleton to allow a more anterior placement of the masseter muscle. Such a placement naturally also enhances bite force on the molars, which might be advantageous for consuming certain types of mechanically challenging foods. However, the bony modifications allowing an anteriorly placed masseter weaken the facial skeleton [2]. Thus, structural reinforcement is needed to compensate for the higher stresses that would otherwise be produced. A key prediction of this hypothesis is that the australopith face is not profoundly stronger than those of non-human apes or hominins that did not experience selection for anteriorly placed masseter muscles. Our findings on *H. habilis* are compatible with this prediction.

In conclusion, our results highlight the centrality of bite force production in Plio-Pleistocene hominin evolution. Australopiths exhibit anteriorly positioned attachments for certain jaw adductor muscles that increase muscle leverage and allow the generation of high bite forces (e.g. [1,3]). They concurrently evolve tall faces/mandibular rami (e.g. [99]) that (in addition to increasing the MA of the jaw adductor muscles [100]) allow them to avoid TMJ distraction while using their balancing side jaw adductor muscles at high activity levels [27,30] during bites on molars (which remain proportionally large distally [86,101], underscoring that the distal-most teeth play a meaningful functional/behavioural role), and they evolve bony structures that reinforce the facial skeleton so as to mechanically compensate for the higher bite forces [2,30]. They also evolve large postcanine teeth (e.g. [102]) with thick enamel (e.g. [103–105]) that make them less liable to fracture under high loads (e.g. [8]). The evolution of these traits comprises many of the most distinctive trends in the evolution of the australopiths, and virtually all of these trends are reversed or otherwise changed in *Homo,* which exhibits a decreased ability to generate molar bite force. The emergence of *Homo,* therefore, marks a profound shift in feeding biomechanics compared with most australopiths that led to major downstream consequences for hominin morphology, perhaps by releasing functional demands on the feeding apparatus (i.e. by removing the need to process foods using high bite forces). The specific dietary variables associated with this shift may be difficult to elucidate, but it is evident that changing selective pressures related to feeding played a meaningful role in the origin of our genus.

## 3. Methods

### 3.1. Model construction

Our FEM of *H. habilis* is based on a virtual reconstruction of the KNM-ER 1813 cranium [106], which was undertaken with finite element analyses of feeding biomechanics specifically in mind. To create the FEM, we first created a completely closed (‘watertight’) stereolithography (STL)-formatted surface mesh of the ER 1813 reconstruction using Geomagic Studio 2014 (Research Triangle Park, NC, USA). Volumes representing the trabecular bone and spaces (as opposed to individual trabeculae) were also generated in Geomagic Studio for the supraorbital region, zygomatic and midface surrounding the tooth roots. During this process, data on craniofacial cortical bone thickness in chimpanzees and gorillas gathered during our analysis of extant ape bone material properties [28] were also used to determine reasonable bone thicknesses across the face. This FEM was compared with our previously constructed models of modern chimpanzees (*P. troglodytes*) [28], recent humans (*H. sapiens*) [29] and a sample of australopiths, including *Australopithecus africanus* (based on Sts 5 and Sts 52) [27,32], *A. sediba* (based on MH1) [5], *A. afarensis* (based on A.L. 444−2) [30] and *P. boisei* (based on OH5) [27]. Our prior research on primate feeding biomechanics has shown that the inclusion of a periodontal ligament (PDL) does not have a major impact on global patterns of cranial bone strain [107]. We therefore chose to not include this structure in our FEMs.

### 3.2. Material properties

Models were assigned spatially heterogeneous isotropic material properties (Young’s modulus [*E*] and Poisson’s ratio [*v*]) of cranial cortical bone from extant taxa (see electronic supplementary material, table S1). Models of fossil hominins and chimpanzees were assigned properties averaged from one chimpanzee and one gorilla from 14 homologous locations across the craniofacial skeleton (average *E* = approx. 17 GPa) [28], while the human models were assigned properties averaged from two modern human specimens from the same 14 sites plus 15 additional sites (average *E* = approx. 21 GPa) [29]. Bone material properties in chimpanzees and human crania are broadly similar [108] and these are the extant taxa that phylogenetically bracket early hominins, so our assumptions about bone material properties are reasonable. In each group, spatially heterogeneous elastic moduli were distributed throughout the cortical volumes of our FEMs using a thermal diffusion technique [109]. This involved first creating a linear relationship between modulus and temperature and seeding the sampled craniofacial sites of the FEM with their respective temperatures. Bone properties were set to be thermally conductive (conductivity = 1), while the effect of temperature on stress and strain was removed (expansion = 0). The temperature distribution was determined by solving the steady-state (non-transient) thermal problem in Strand7 (Strand7 Pty Ltd, NSW, Sydney, Australia). Elastic moduli were then set to vary with temperature during the loading analyses described below. Volumes of trabecular bone and those for the tooth crowns were assigned homogeneous isotropic material properties of 0.637 and 80 GPa, respectively, each with a Poisson’s ratio of 0.3, following our previous work [2,5,27–30,81,95].

### 3.3. Muscle force scaling and loading conditions

The scaling of muscle force magnitudes in analyses that involve models of different sizes is a crucial issue in comparative biomechanics. Here and in our previous mechanical analyses of fossil hominins and extant hominoids [2,5,27–30,81,95], we have removed the effects of size from the stress and strain results by scaling the jaw adductor muscle forces (anterior temporalis, superficial masseter, deep masseter, medial pterygoid) applied to each model by a proxy for size, volume^2/3^, using baseline forces calculated from physiological cross-sectional areas (PCSAs) in an adult female chimpanzee [32] (see electronic supplementary material, table S2). Combined with the assignment of identical sets of material properties, this procedure focuses the mechanical results on differences in shape alone (i.e. it removes the confounding effects of size) [31,95] and provides a powerful means of assessing the relative strength of each model. However, in the case of *H. habilis*, scaling from baseline chimpanzee muscle forces almost certainly overestimates bite force magnitudes and strain levels, whereas modern human muscle forces probably underestimate its biting capabilities. We therefore examined bite force generation separately by loading the chimpanzee and australopith crania with isometrically scaled muscle forces derived from a chimpanzee [32], and by loading the modern human crania with isometrically scaled muscle forces derived from a human [29]. The FEM of *H. habilis* was loaded twice for each bite case, using baseline forces scaled from either chimpanzees or humans (see electronic supplementary material, table S2).

Some models, including modern humans, *H. habilis*, and *A. sediba*, exhibited tensile (distractive) forces at the working (biting) side TMJ, which, in life, would have ‘pulled’ apart the soft tissues of the TMJ capsule and increased the risk of joint dislocation during powerful molar biting [44,72]. Therefore, we determined the balancing (non-biting) side muscle force reduction required to eliminate joint tension by iteratively reducing the forces by 5% until the working side joint returned to compression. In *H. habilis*, joint tension was relatively high, requiring large reductions in balancing side muscle force (45% when applying chimpanzee forces, 20% when applying human forces; table 2).

Scaled muscle forces for the anterior temporalis, superficial masseter, deep masseter and medial pterygoid were applied to the cranial origins of each model using a software package (Boneload) that accounts for the increased torque produced when muscles wrap around curved bone surfaces [110]. Muscle force vectors were oriented towards their respective insertion sites on the mandible, defined as the three-dimensional area centroid of each muscle’s insertion area using a different software package (Area Centroids), with the mandible of each FEM slightly depressed and with the condyles translated onto the articular eminences. No suitable mandible of *H. habilis* (i.e. one that preserves the muscle insertion sites) is available, so the rami for MH1 and one modern human (GRGL) were each scaled to best fit ER 1813. The use of these rami produced nearly identical results, so the centroid for each muscle’s insertion area was averaged between them.

Mechanical analyses of biting were performed in Strand7 (Strand7 Pty Ltd, NSW, Sydney, Australia). During each load case, models were oriented to a line best fit to the postcanine occlusal plane and an axis of rotation was created by constraining the TMJ against translation at the working (all directions) and balancing (vertical and anteroposterior directions) sides. For the premolar simulation, a node in the centre of the occlusal surface of the left upper third premolar (P^3^) was constrained in the vertical direction, while the left upper second molar (M^2^) was similarly constrained for the molar biting simulation. Upon the application of muscle forces, these constraints permit the cranium to rotate about the TMJ axis, ‘pulling’ it down onto the bite point, generating stress and strain in the craniofacial skeleton and reaction forces at the constrained nodes. Prior work [29,32,111,112] has shown that primate cranial FEMs constructed using the methods employed here deform realistically and simulate realistic bite forces.

### 3.4. Analysis of model output parameters

We displayed global von Mises microstrain patterns using strain maps, which provide information on both the magnitude and spatial patterning of strain distributions. These maps are analogous to histograms in that they illustrate strain magnitudes at thousands (or millions) of integration points simultaneously. We also compared data on von Mises strain magnitude from 12 functionally homologous locations across the craniofacial skeleton. These locations correspond to those included in our prior research on fossil hominin feeding biomechanics [2,5,27–30,81,95] and include those from previous *in vitro* bone strain analyses in extant primates [112,113]. The ER 1813 specimen, from which our model of *H. habilis* was built, is missing both of its zygomatic arches, so it was reconstructed based on those in Sts 5 [106]. We therefore did not compare strains from the zygomatic arch.

Bite force in our analysis was quantified in newtons (N) using the reaction force at the constrained bite point, which measures a compressive force normal to the postcanine occlusal plane. Bite force leverage (i.e. efficiency) for all load cases was quantified using the mechanical advantage (MA), calculated as the ratio of bite force output to muscle force input. Reaction forces at the jaw joints were analysed within the context of the constrained lever model of feeding biomechanics [44,72] (see electronic supplementary material). This model predicts that species in need of high bite force should exhibit craniofacial adaptations that ensure stable (i.e. compressive) reaction forces at both TMJs. This occurs during anterior tooth (including premolar) biting because the muscle resultant vector of the jaw adductors on both sides of the head pass through a ‘triangle of support’ formed by the bite point and two articular eminences. However, biting on the distal teeth increases the risk of generating distractive (tensile) reaction forces at the working (biting) side joint that ‘pull’ apart the soft tissues of the joint capsule and increase the risk of joint dislocation. These tensile forces can be mitigated through reductions in balancing (non-biting) side muscle force. In our models, TMJ reaction forces were recorded relative to a user-defined ‘triangle of support’ Cartesian coordinate system, with one of three axes perpendicular to a reference plane defined by the three constrained nodes (i.e. the ‘triangle of support’), meaning this coordinate system differed during P^3^ and M^2^ biting.

## Data Availability

Our data, in the form of FEMs, are available online [114]. Supplementary material is available online [115].
